# Nanodiamond Integration into Niosomes as an Emerging and Efficient Gene Therapy Nanoplatform for Central Nervous System Diseases

**DOI:** 10.1021/acsami.2c02182

**Published:** 2022-03-15

**Authors:** Nuseibah AL Qtaish, Idoia Gallego, Alejandro J. Paredes, Ilia Villate-Beitia, Cristina Soto-Sánchez, Gema Martínez-Navarrete, Myriam Sainz-Ramos, Tania B. Lopez-Mendez, Eduardo Fernández, Gustavo Puras, José Luis Pedraz

**Affiliations:** † NanoBioCel Research Group, Laboratory of Pharmacy and Pharmaceutical Technology, Faculty of Pharmacy, University of the Basque Country (UPV/EHU), Paseo de la Universidad 7, 01006 Vitoria-Gasteiz, Spain; ‡ Networking Research Centre of Bioengineering, Biomaterials and Nanomedicine (CIBER-BBN), Institute of Health Carlos III, 28029 Madrid, Spain; § Bioaraba, NanoBioCel Research Group, 01009 Vitoria-Gasteiz, Spain; ∥ Research and Development Unit in Pharmaceutical Technology (UNITEFA), CONICET and Department of Pharmaceutical Sciences, Chemistry Sciences Faculty, National University of Córdoba, Haya de la Torre y Medina Allende, X5000XHUA Córdoba, Argentina; ⊥ School of Pharmacy, Queen’s University Belfast, Medical Biology Centre, 97 Lisburn Road, Belfast BT9 7BL, Northern Ireland, U.K.; # Neuroprothesis and Neuroengineering Research Group, Institute of Bioengineering, Miguel Hernández University, Avenida de la Universidad, 03202 Elche, Spain

**Keywords:** nanodiamonds, niosomes, cationic lipids, gene delivery, nanomedicine, CNS diseases

## Abstract

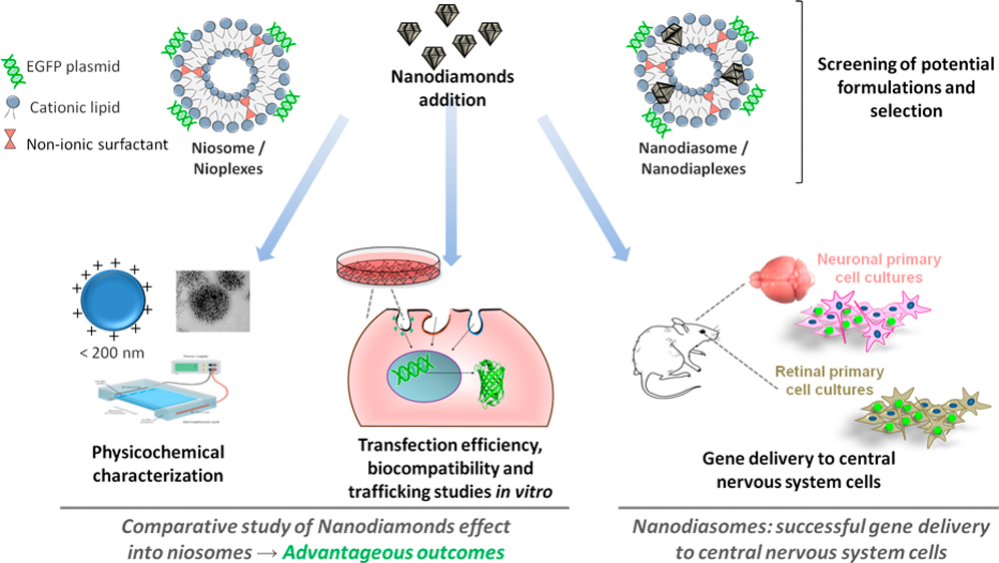

Nanodiamonds (NDs) are promising materials for gene delivery because of their unique physicochemical and biological features, along with their possibility of combination with other nonviral systems. Our aim was to evaluate the biophysical performance of NDs as helper components of niosomes, named nanodiasomes, to address a potential nonviral gene delivery nanoplatform for therapeutic applications in central nervous system (CNS) diseases. Nanodiasomes, niosomes, and their corresponding complexes, obtained after genetic material addition at different ratios (w/w), were evaluated in terms of physicochemical properties, cellular uptake, intracellular disposition, biocompatibility, and transfection efficiency in HEK-293 cells. Nanodiasomes, niosomes, and complexes fulfilled the physicochemical features for gene therapy applications. Biologically, the incorporation of NDs into niosomes enhanced 75% transfection efficiency (*p* < 0.001) and biocompatibility (*p* < 0.05) to values over 90%, accompanied by a higher cellular uptake (*p* < 0.05). Intracellular trafficking analysis showed higher endocytosis via clathrins (*p* < 0.05) in nanodiaplexes compared with nioplexes, followed by higher lysosomal colocalization (*p* < 0.05), that coexisted with endosomal escape properties, whereas endocytosis mediated by caveolae was the most efficient pathway in the case of nanodiaplexes. Moreover, studies in CNS primary cells revealed that nanodiaplexes successfully transfected neuronal and retinal cells. This proof-of-concept study points out that ND integration into niosomes represents an encouraging nonviral nanoplatform strategy for the treatment of CNS diseases by gene therapy.

## Introduction

For more than 50 years, it has been hypothesized by the scientific community that therapies based on the delivery of genetic materials could be an appealing option to face human diseases. In theory, this strategy, so-called gene therapy, would offer the possibility of achieving durable and curative clinical benefit. At present, this approach is widely applied in clinical trials, with some of them recently achieving approved drug status in the United States and Europe.^1^ Nevertheless, this approach is still far from being considered a mainstream therapeutic option, as vectors used have not demonstrated the desirable characteristics in terms of safety, efficacy, or associated costs.

The most basic form of gene therapy is naked plasmid DNA; however, its poor cellular uptake, degradation by nucleases, and low transfection efficiency make necessary the use of vectors able to protect and suitably deliver the nucleic acids.^2^ At present, most DNA delivery strategies use viral or nonviral vectors. Although viral vectors such as lentiviruses,^3^ adenoviruses,^4^ and recombinant adeno-associated viruses^5^ provide higher efficiency over a longer period, there are important limitations concerning safety issues, including toxicity, immunogenicity, mutagenesis, and inflammatory potential, as well as high production costs.^6^ These limitations have boosted the need to develop safer and less cytotoxic nucleic acid carriers, as is the case of nonviral systems.^7^ Research on chemical nonviral vectors has gained momentum as they are comparatively less invasive than viral ones, show less immune and inflammatory responses, are cheaper to produce, and have higher genetic material cargo capacity.^8^ However, their low transfection efficiency represents the most important handicap for clinical applications. Therefore, the scientific community continues to seek novel strategies able to overcome this obstacle.

Nanomaterials, such as carbon atom-based molecules, have captured the attention in the field of nanotechnology intended for biomedical applications. In particular, nanodiamonds (NDs) constitute an attractive platform for drug and gene delivery because of their unique physicochemical features, biocompatibility, near-spherical shape, narrow particle size distribution, water dispersibility, high specific area, and ease of surface functionalization.^9,10^ In particular, some authors have vectored plasmid DNA^11,12^ or siRNA^13−15^ by NDs after functionalization with polyethylenimine 800, polyglycerol, lysine, or polyallylamine hydrochloride through the formation of electrostatic bonded complexes. In contrast, other studies have achieved those deliveries by covalent derivatization of NDs with silane-NH_2_ groups^11,16^ and polyamidoamine^17^ or by joining NDs to EDA (joint arm) and H-Arg-GlyAsp-Val-OH (targeting agent).^18^ Nevertheless, some of the limitations of these carbon-based nanostructures include their need of binding to other vectors for their stabilization and the low gene packing capacity achieved to date with the conventional linkers. Hence, there arises the need for developing other systems able to overcome the concerns related to NDs.

In this sense, lipidic vectors such as niosomes are high DNA packing gene delivery systems that offer the ability to condense, protect, and suitably release DNA in a safe manner, making them a widely used prime candidate for nonviral gene therapy.^19−21^ Basically, niosomes for gene delivery are composed of a cationic lipid to promote electrostatic interactions with negatively charged molecules^22^ and nonionic surfactants to enhance the stability,^23^ and there exists the possibility to include a helper component that would improve the biological activity of the vector.^24,25^ Although the main limitation of this kind of vector is its lower transfection efficiency compared to viral ones, we hypothesize that the incorporation of emerging nanomaterials like NDs as a helper component into the structure of niosomes could potentially improve this ability and might lead to a powerful gene delivery tool for translational therapeutic applications, and particularly for central nervous system (CNS) diseases, where the blood–brain and blood–retinal barriers hamper even more the implementation of therapeutic strategies.^26^

Therefore, and in the absence of any evidence related to the incorporation of NDs into niosomes, the aim of this study was to combine NDs with the components used for the preparation of niosomes to develop an optimized nonviral vector-based nanoplatform for efficient and safe gene therapy with potential translation into biomedical application. To this end, we employed monodispersed ND particles, the cationic lipid 1,2-di-O-octadecenyl-3-trimethylammonium propane (DOTMA), and the nonionic surfactant polysorbate Tween 20, obtaining NDs integrated into niosomes, named nanodiasomes, and niosomes devoid of NDs. These vectors were combined with pEGFP plasmid to form the corresponding nanodiaplexes and nioplexes, respectively; all of them were physicochemically characterized concerning particle size, zeta potential, dispersity, and morphology and were assessed in terms of capacity to condense, protect, and release the DNA from enzymatic digestion. The biological performance of NDs into niosomes was additionally analyzed by in vitro assays to determine the biocompatibility and transfection efficiency of this gene delivery system in the HEK-293 cell line, as well as the cellular uptake and intracellular disposition of nanodiaplexes versus nioplexes. Finally, experiments in rat CNS primary cells, from neuronal and retinal origin, were performed to assess the gene delivery ability of this novel nanoplatform in a more closer to reality biological scenario aimed at treating CNS diseases by gene therapy.

## Experimental Section

### Elaboration of Formulations

**Figure 1:**
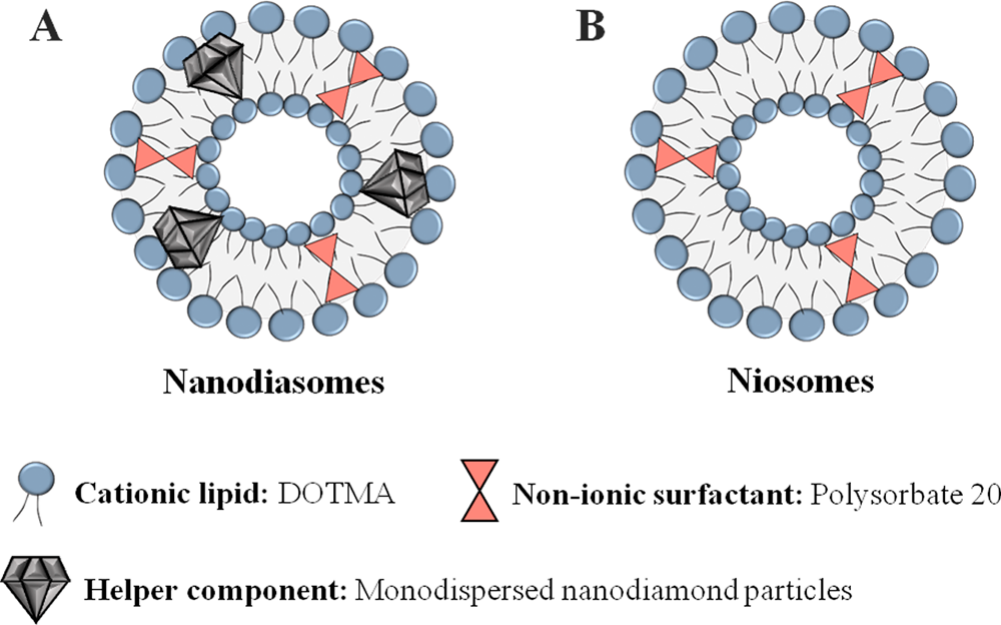
Overview of the components and their disposition in (A) nanodiasomes and (B**)** niosomes.

All the formulations were elaborated by the oil-in-water emulsion technique. NDs were purchased as ultrananocrystalline diamonds with particle size smaller than 10 nm (Sigma-Aldrich Madrid, Spain, ID: 900180). A volume of 250 μL of NDs (10 mg/mL in H_2_O) was ultrasonicated for 30 min and mixed with 2 mL of 0.5% Tween 20 (Sigma-Aldrich Madrid, Spain) and 1.75 mL of MilliQ water, as the aqueous phase. On the other hand, 1.25, 2.5, or 5 mg of the cationic lipid DOTMA (Avanti Polar Lipids, Inc., Alabama, USA) were accurately weighed to obtain 1/0.5, 1/1 and 1/2 ND/DOTMA mass ratios, respectively. The DOTMA was diluted in 1 mL of dichloromethane (DCM) (Panreac, Barcelona) which constituted the organic phase. This phase was added upon the aqueous phase and immediately sonicated for 30 s at 50 W (Branson Sonifier 250, Danbury). DCM was evaporated for 2 h at room temperature (RT) under magnetic stirring obtaining formulations named nanodiasomes NDT10, NDT11, and NDT12, for ND/DOTMA at 1/0.5, 1/1, and 1/2 mass ratios, respectively. The elaboration of niosomes, as control formulations with no ND, was carried out following the same abovementioned protocol using the same amounts of DOTMA in the organic phase. Figure 1 shows the components employed for their elaboration of both formulations, as well as a schematic representation of the distribution of these components in nanodiasomes (Figure 1A) and niosomes (Figure 1B).

### Preparation of Complexes

Complexes, named nanodiaplexes and nioplexes, were prepared by mixing nanodiasomes or niosomes with propagated pEGFP plasmid following a previously reported methodology,^25^ to obtain complexes at 2/1, 5/1, 10/1, and 15/1 cationic lipid/DNA mass ratios.

### Physicochemical Characterization of Formulations and Complexes

The mean particle size and dispersity index (*Đ*) of nanodiasomes, niosomes, and their corresponding nanodiaplexes and nioplexes were determined by cumulative analysis as previously described.^21^

### Morphological Characterization

To assess the shape and morphology of the formulations, transmission electron microscopy (TEM) was employed as previously described.^27^ To analyze the disposition of NDs in the nanodiasomes, further microscopy studies were performed by means of cryo-tomography. For that, 1 mg/mL sample was diluted to 0.5 mg/mL with bovine serum albumin (BSA)-gold nanoparticles (10 nm) required for accurate tomographic tilt series alignment (https://aurion.nl/products/aurion-gold-tracers/). After vortex shaking, 3 μL of the sample were applied to the Cu/Rh R2/2 Quantifoil grid and vitrified using ThermoFisher Scientific Vitrobot Mark IV at 22 °C 95% humidity.

Vitrified samples were entered in a Talos Arctica (ThermoFisher Scientific, Spain) operated at liquid nitrogen temperature (200 Kv). The dose symmetric tilt scheme^28^ was used to acquire tilted series to a final total dose of 130 e-Å^2^ using Tomography software from ThermoFisher Scientific (step 3°, ±65° at 28.000× with a pixel size of 1.44 nm/pix). Tilt series alignment was performed using IMOD^29^ and reconstruction with SIRT using TOMO3D.^30^ Reconstructed volumes were analyzed with ImageJ^31,32^ and 3D rendering was performed with USFC Chimera.^33^

### Gel Retardation Assay

The capacity of both complexes at different cationic lipid/DNA mass ratios to condense, protect, and release the genetic material was assessed by a 0.8% (w/w) agarose (Sigma-Aldrich, Spain) gel electrophoresis assay. To analyze the DNA binding capacity of formulations, samples were directly loaded into the gel. To evaluate the DNA protection capacity of formulations, 4 μL of DNase I enzyme (Thermo Fisher Scientific, Spain) were added and incubated for 30 min at 37 °C, and then, 6 μL of 10% sodium dodecyl sulfate (SDS) (Sigma-Aldrich, USA) were added and incubated for 10 min at RT. To examine the DNA release from the complexes, the same quantity of SDS was added to the samples and incubated for 10 min at RT. After the addition of 4 μL of loading buffer per sample, the agarose gel was immersed in a Tris-acetate-EDTA buffer and subjected to electrophoresis for 30 min at 120 V. Naked DNA was used as a control, 200 ng being the amount of DNA used per well in all cases. DNA bands were stained with GelRed reagent and observed under a ChemiDoc MP Imaging System, for further analysis by Image Lab Software (BioRad, USA).

### Cell Culture and In Vitro Transfection Assays

Human embryonic kidney cells 293 (HEK-293; ATCC, CRL1573) were cultured and maintained as previously described.^27^ To carry out transfection experiments, HEK-293 cells were seeded in 24-well plates at a density of 20 × 10^4^ cells per well in medium without antibiotics and incubated overnight to achieve 70% of confluence. Nanodiaplexes and nioplexes at different cationic lipid/DNA mass ratios were prepared by their incubation with pEGFP in OptiMEM transfection medium (Gibco, San Diego, CA, USA) for 30 min at RT. After removing the growth medium, cells were exposed to these complexes for transfection during 4 h in an incubator. Hereinafter, complexes were removed, and fresh medium was added. Positive and negative controls of transfection were performed using Lipofectamine 2000 (Invitrogen, Carlsbad, CA, USA) and nontreated cells were exposed only to OptiMEM for 4 h, respectively. Each condition was performed in triplicate.

Transfection efficiency was reported, both qualitatively and quantitatively, 48 h after the addition of complexes by fluorescence microscopy (EclipseTE2000-S, Nikon) and the flow cytometry technique (FACSCalibur, Becton Dickinson Bioscience, San Jose, USA), respectively. In the latter case, after a rapid wash step, HEK-293 cells were exposed to 300 μL of 0.05% trypsin/EDTA for detachment. Later, growth medium was added to block the trypsin effect. Thereafter, cells were centrifuged to obtain the cell pellet eliminating the supernatant. Next, the pellet was resuspended with phosphate buffered saline (PBS) and diluted in FACSFlow liquid. Such cells were placed in flow cytometer tubes to quantify the EGFP signal in living cells. Cell viability was evaluated by staining cells with propidium iodide (Sigma-Aldrich, USA) before performing flow cytometry. The fluorescent emission of both dead and transfected cells was evaluated at 650 nm (FL3) and 525 nm (FL1), respectively. The mean fluorescence intensity (MFI) signal was analyzed from live positive cells in the FL1 channel. The collection gate was established employing nontransfected cells. Flow cytometer settings and channel compensation were performed using cells transfected with Lipofectamine 2000. Cell viability and transfection data were normalized considering the values of negative and positive control cells, respectively. The experiments were carried out in triplicate, collecting a minimum of 10,000 events for each sample. FlowJo software (Becton Dickinson) was used to analyze the data.

### Cellular Uptake

The uptake of nanodiaplexes and nioplexes was analyzed by incubating cells with fluorescein isothiocyanate (FITC)-labeled pEGFP (FITC-pEGFP) for 4 h. The FITC positive signal was analyzed both, qualitatively and quantitatively. For qualitative assays, cells were seeded on coverslips to fix them with 4% formaldehyde (Panreac, Spain) after the incubation. Once fixed, cells were washed with PBS and exposed to phalloidin (5 μL) in PBS with 1% BSA for 40 min to stain their cytoskeleton. After a PBS washing step, cells were mounted with Fluoroshield with DAPI (Sigma-Aldrich, USA). Afterward, mounted cells were analyzed with confocal laser scanning microscopy (Zeiss Axiobserver). Images were examined with ImageJ software. Quantitative analysis was carried out by flow cytometry as described before. Cellular uptake data were normalized to positive control cells treated with Lipofectamine 2000 and expressed as the percentage of FITC-pEGFP positive cells.

### Intracellular Trafficking

Cellular internalization of nanodiaplexes and nioplexes was analyzed by incubating cells with FITC-labeled pEGFP (FITC-pEGFP) for 3 h over coverslips as described before. Afterward, specific endocytic pathway markers were coincubated for 1 h: transferrin-AlexaFluor594 (50 μg/mL) to stain clathrin-mediated endocytosis (CME), cholera toxin B-AlexaFluor594 (10 μg/mL) to stain caveolae mediated endocytosis (CvME), dextran-AlexaFluor594 (1 μg/μl) for macropinocytosis, or lysotracker Red-DND-99 (20 μM) for the lysosomal late endosomal compartment. After fixation of cells and mounting, slides were observed under microscopy to capture representative images for their analysis by the ImageJ software. Green and red signal colocalization, corresponding to the endocytic pathway and to FITC-pEGFP, respectively, was measured by cross-correlation analysis.^34,35^

Additionally, specific endocytosis inhibitors were used to inhibit cellular uptake prior to the transfection assay. For this, in a 24-well plate, HEK-293 cells were exposed for 30 min with 200 μM genistein, and for 60 min with 5 μg/mL chlorpromazine hydrochloride and with 50 nM wortmannin (Thermo Fisher Scientific, Madrid, Spain), as inhibitors for CvME, CME, and macropinocytosis pathways, respectively. Then, the medium containing the inhibitors was removed, a rapid wash was performed, and transfection was carried out with both nioplexes and nanodiaplexes vectoring pEGFP plasmid, as described before. Cells were processed, and EGFP-positive cells were quantitatively assessed by flow cytometry as detailed before. Data were normalized in relation to the value of EGFP-positive cells after transfection with nanodiaplexes and nioplexes and with no inhibitors of the endocytic pathways. The experiments were carried out in triplicate collecting and analyzing more than 5000 events for each sample.

### Endosomal Escape of the Complexes from the Late Endosome

Anionic micelles based on phosphatidylserine (PS) (Sigma-Aldrich, Spain) were prepared to mimic the late endosomal compartment. Chloroform at 1.6 mM was used to dissolve PS and exposed to magnetic agitation to evaporate the solvent. The dried sample was reconstituted in PBS and sonicated to obtain a dispersed solution containing PS micelles. Nanodiaplexes and nioplexes were incubated, or not, with the PS micelles for 1 h at 1:50 pEGFP:PS mass ratio. Samples, containing 200 ng of DNA, were loaded in a 0.8% agarose gel and subjected to electrophoresis to observe the amount of genetic material released from the complexes. The electrophoresis process, band staining, and analysis were carried out as previously mentioned in the Gel Retardation Assay section.

### Animals, Procedures, and Exposure to Nanodiaplexes

Procedures carried out with animals for scientific research purposes were performed following the RD 53/2013 Spanish and 2010/63/EU European Union regulations, and according to the Miguel Hernandez University Standing Committee for Animal Use in the Laboratory. Primary CNS cells were extracted from the brain cortex and retinal tissue of E17–E18 rat embryos (Sprague Dawley) and processed as described elsewhere.^36,37^ Lipofectamine 2000 (Invitrogen, California, USA) at 2/1 ratio was employed as a positive control. Each condition was performed in triplicate.

### Evaluation of Gene Transfection in CNS Primary Cells

EGFP expression from primary neuronal and retinal transfected cells was examined 72 h after their exposure to nanodiaplexes to qualitatively assess the transfection efficiency by immunocytochemistry.^36^ Briefly, cover slips were incubated overnight with chicken anti-EGFP (Invitrogen, 1:300). Cells were incubated for 1 h with secondary antibody Alexa Fluor555 goat anti-chicken IgG (Invitrogen, 1:100) which was pseudocolored in green to visualize EGFP expression. Nuclei were stained with Hoechst 33342 (Sigma-Aldrich, Spain). Confocal images were obtained using a laser-confocal microscope (Leica TCS SPE Microsystems GmbH, Germany).

### Statistical Analysis

Normality and homogeneity of variances were confirmed by the Shapiro–Wilks and the Levene tests, respectively. Then, a one-way analysis of variance followed by the Student–Newman–Keuls test was performed to analyze the differences between more than two groups. Under nonparametric conditions, the Kruskal–Wallis test followed by a Mann–Whitney *U* test was employed. Differences between two groups for unpaired data were analyzed using Student’s *t*-test or a Mann–Whitney *U* test, as appropriate. Data were expressed as mean ± standard deviation (SD). A *p* value < 0.05 was considered statistically significant. SPSS 15.0 statistical software was used to analyze data.

## Results

### Biophysical Screening of Nanodiasome Formulations

Three nanodiasome formulations with different DOTMA compositions, named NDT10, NDT11, and NDT12, were elaborated (Supporting Information) and evaluated in terms of physicochemical properties (Figure S1, Supporting Information), as well as transfection ability and cytotoxicity (Figure S2, Supporting Information). This screening of formulations led to the conclusion that the nanodiasome with better biophysical performance for gene delivery purposes was the NDT12, which corresponds to the 1/2 ND/DOTMA mass ratio formulation. In consequence, this NDT12 nanodiasome formulation and its respective niosome control, devoid of NDs, were employed for further studies.

### Physicochemical Characterization of Formulations and Complexes

**Figure 2:**
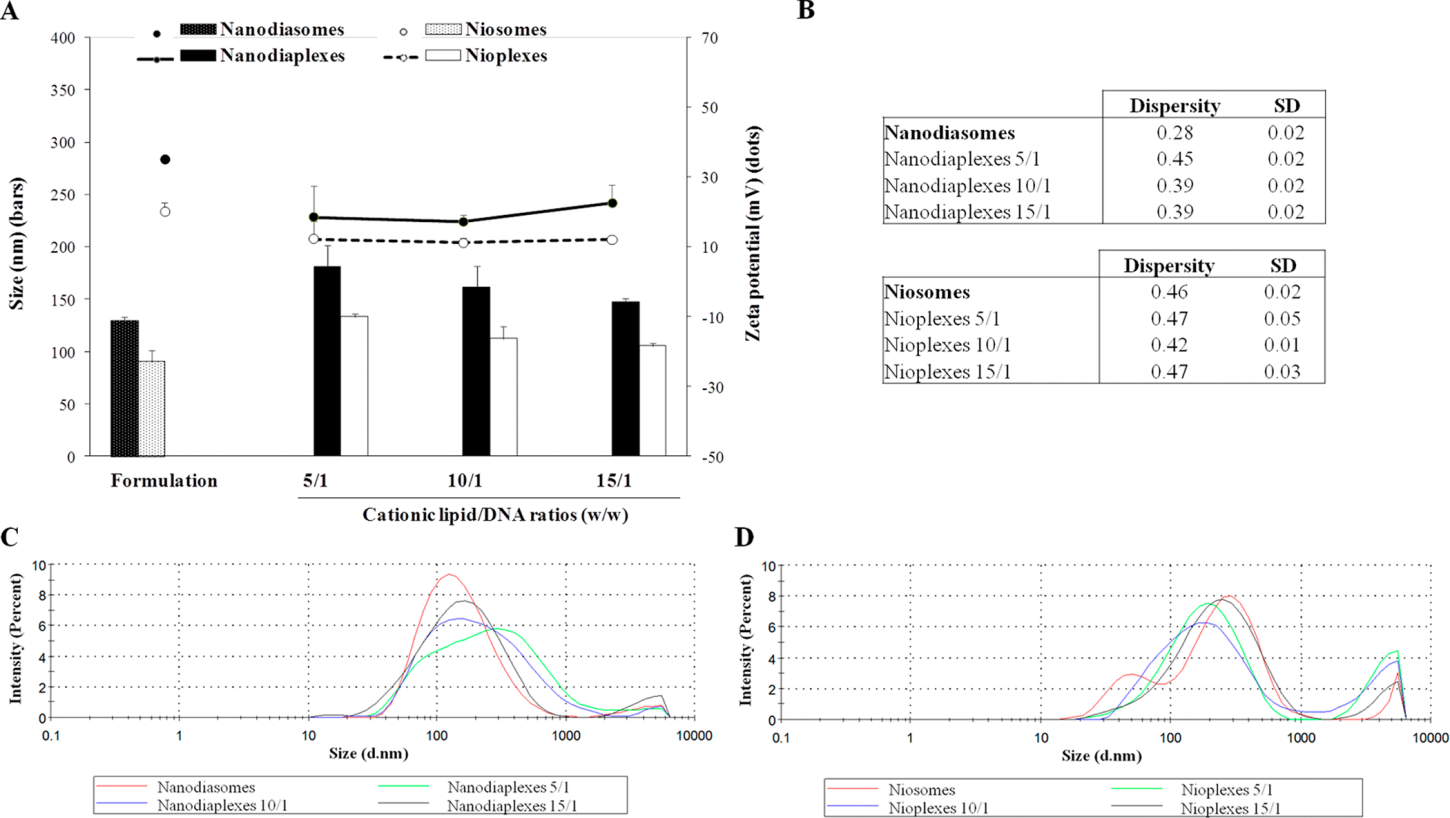
Characterization of formulations and complexes prepared with NDs (nanodiasomes/nanodiaplexes) and without NDs (niosome/nioplexes). (A) Size (bars) and zeta potential (dots). (B) Dispersity values of formulations and complexes. Each value represents the mean ± SD of three measurements. (C) Average size-distribution intensities of nanodiasomes (red line) and nanodiaplexes at different cationic lipid/DNA mass ratios (green, blue and black line for 5/1, 10/1, and 15/1 ratios, respectively). (D) Average size-distribution intensities of niosomes (red line) and nioplexes at different lipid/DNA ratios (green, blue, and black line for 5/1, 10/1, and 15/1 ratios, respectively).

The particle size of formulations and their corresponding complexes was below 200 nm in all cases (Figure 2A, bars). In particular, nanodiasome and nanodiaplexes presented nearly a 30% higher particle size than niosomes and nioplexes. Upon the addition of pEGFP to formulations, the mean particle size values slightly increased around 40% at the 5/1 ratio and gradually decreased when increasing the lipid/DNA ratio in both complexes. Zeta potential values for nanodiasomes were above +30 mV, precisely 35.2 ± 0.3, while those for niosomes were below this number, with a value of 20.2 ± 2.5 (Figure 2A, dots). After plasmid condensation, zeta potential of nanodiaplexes and nioplexes at the 5/1 lipid/DNA ratio decreased moderately and increased slightly when augmenting the lipid/DNA ratios, especially in the case of nanodiaplexes (Figure 2A, dots). Regarding dispersity (*Đ*) (Figure 2B), values for nanodiasomes and nanodiaplexes were in general below 0.4, while those for niosomes and nioplexes were above 0.4. Dynamic light scattering size-distribution profiles of niosomes, nanodiasomes, and their complexes can be observed in Figure 2C,D.

### Morphological Characterization

**Figure 3:**
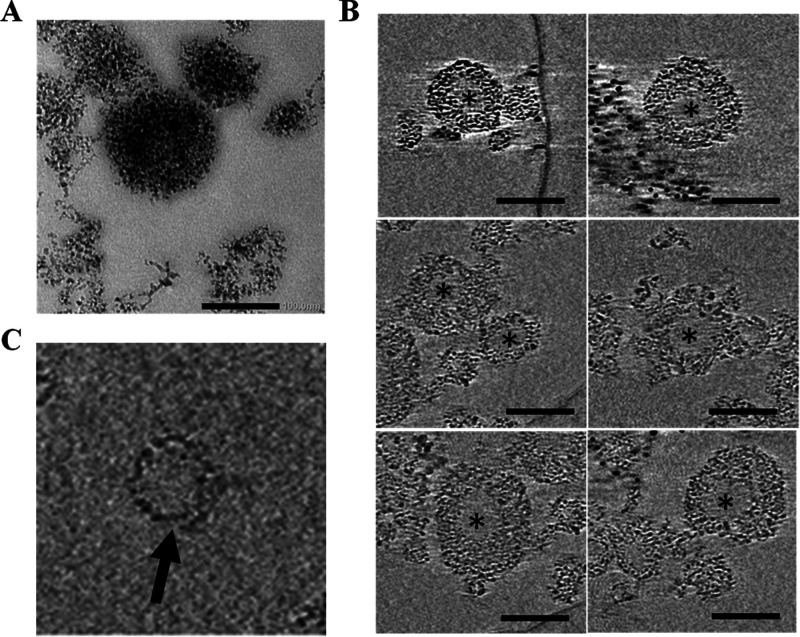
Microscopy images of nanodiasomes. (A) TEM image of nanodiasomes. Scale bar: 100 nm. (B) Cryo-TEM images of nanodiasomes; asterisks indicate the aqueous phase. Scale bar: 100 nm. (C) Lipid layer of nanodiasomes (black arrow) with NDs integrated into the lipid structure.

Nanodiasomes observed under TEM (Figure 3A) presented a clear spherical morphology. To go in depth into the disposition of ND particles in the niosome structure to form nanodiasomes, cryo-tomography studies were performed. As observed in Figure 3B, NDs were integrated into the lipid layer of niosomes (Figure 3B,C), rather than on their surface or in their inner aqueous phase (Figure 3B, asterisks). Cryo-tomography reconstruction and the volumetric representation of the tomograms can be observed in the Supporting Information document (Videos 1 and 2 Supporting Information, respectively).

**Figure 4:**
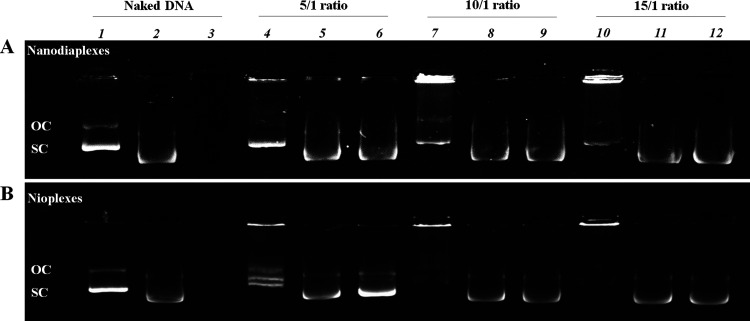
Agarose gel electrophoresis assay. (A) Nanodiaplexes. (B) Nioplexes. Lanes 1–3 correspond to free DNA; lanes 4–6, 5/1 ratio; lanes 7–9, 10/1 ratio; lanes 10–12, 15/1 ratio. Nanodiaplexes and nioplexes were treated with SDS (lanes 2, 5, 8, and 11) and DNase I + SDS (lanes 3, 6, 9, and 12). OC: open circular form; SC: supercoiled form.

To determine the capacity of nanodiasomes to condense, protect, and release the DNA material in comparison with niosomes devoid of ND, a gel retardation assay was performed (Figure 4). Nioplexes (Figure 4B) showed a greater ability than nanodiaplexes (Figure 4A) to bind the DNA, at 10/1 and 15/ratios, because no SC bands were visualized on lanes 7 and 10, respectively. At the lower 5/1 ratio, neither nioplexes nor nanodiaplexes were able to condense all DNA on their surfaces. As expected, no condensation was observed in the control naked DNA (lane 1), which in fact migrated completely in the gel. In this assay, SDS was added to the complexes to mimic a gene delivery microenvironment and promote the release of all the cargo to the media. It was observed that the DNA was released after the addition of SDS to nanodiaplexes and nioplexes at 5/1, 10/1, and 15/1 ratios (lanes 5, 8, and 11, respectively); additionally, it was also protected from DNase I enzymatic digestion at all ratios (lanes 6, 9, and 12) for both nanodiaplexes (Figure 4A) and nioplexes (Figure 4B). The absence of a band on lane 3 demonstrated that naked DNA suffered from DNase I enzymatic digestion.

### Cytotoxicity and Transfection Efficiency In Vitro

**Figure 5:**
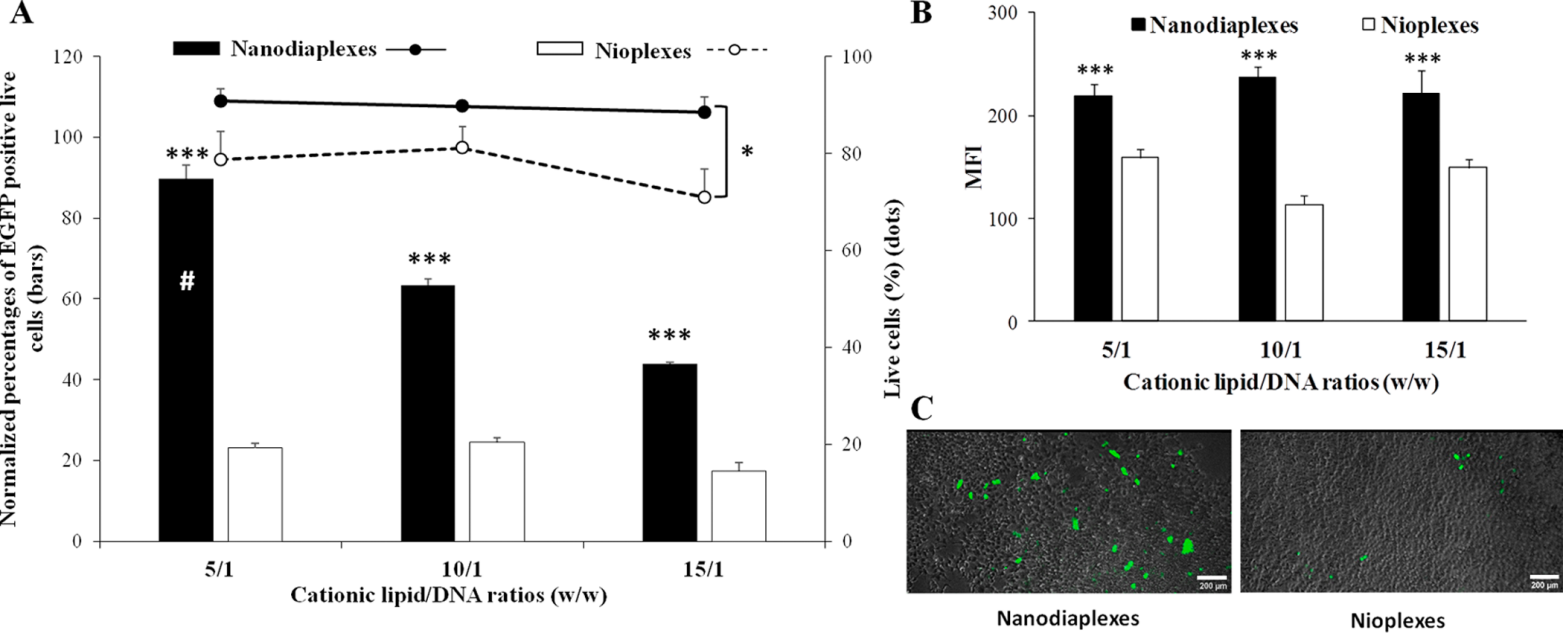
Transfection assay in the HEK-293 cell line 48 h post-transfection with nanodiaplexes and nioplexes. (A) Normalized percentages of EGFP-positive live cells (bars) and cell viability (dots). (B) MFI values. (C) Images showing the EGFP signal and cell integrity in HEK-293 cells transfected with nanodiaplexes and nioplexes at the 5/1 lipid/DNA ratio. Scale bar: 200 μm. Each value represents the mean ± SD of three measurements. ***p < 0.001 and *p < 0.05 for nanodiaplexes vs nioplexes at the same lipid/DNA ratio; #*p* < 0.001 compared with all conditions.

Transfection with nanodiaplexes showed high biocompatibility presenting cell viability values around 90% at all lipid/DNA ratios, while this parameter declined significantly (*p* < 0.05) below 80% when transfecting with nioplexes (Figure 5A, dots). Nanodiaplexes at the 5/1 lipid/DNA ratio were the condition with the highest percentage of EGFP-positive live cells, with a value of 89.1 ± 7.7% (*p* < 0.001). This transfection efficiency supposes a 75% of increment (*p* < 0.001) in comparison with its counterpart nioplexes devoid of ND (22.7 ± 2.4%). This greater pEGFP expression of nanodiaplexes over nioplexes was also observed at 10/1 (62.7 ± 2.7 vs 23.9 ± 2.5%; *p* < 0.001) and 15/1 (43.2 ± 1.1 vs 16.8 ± 4.7%; *p* < 0.001) ratios (Figure 5A, bars). Lipofectamine was employed as a positive control for transfection, which presented a 43% of EGFP expression in live cells (data not shown). All data were normalized in relation to this percentage value.

The superior ability of nanodiaplexes over nioplexes for gene delivery purposes was further corroborated by the MFI assay of the EGFP signal (Figure 5B), with significant differences at all lipid/DNA ratios (*p* < 0.001). Representative fluorescence microscopy images of the EGFP signal in the transfected HEK-293 cell line at the 5/1 ratio can be observed in Figure 5C.

### Cellular Uptake

**Figure 6:**
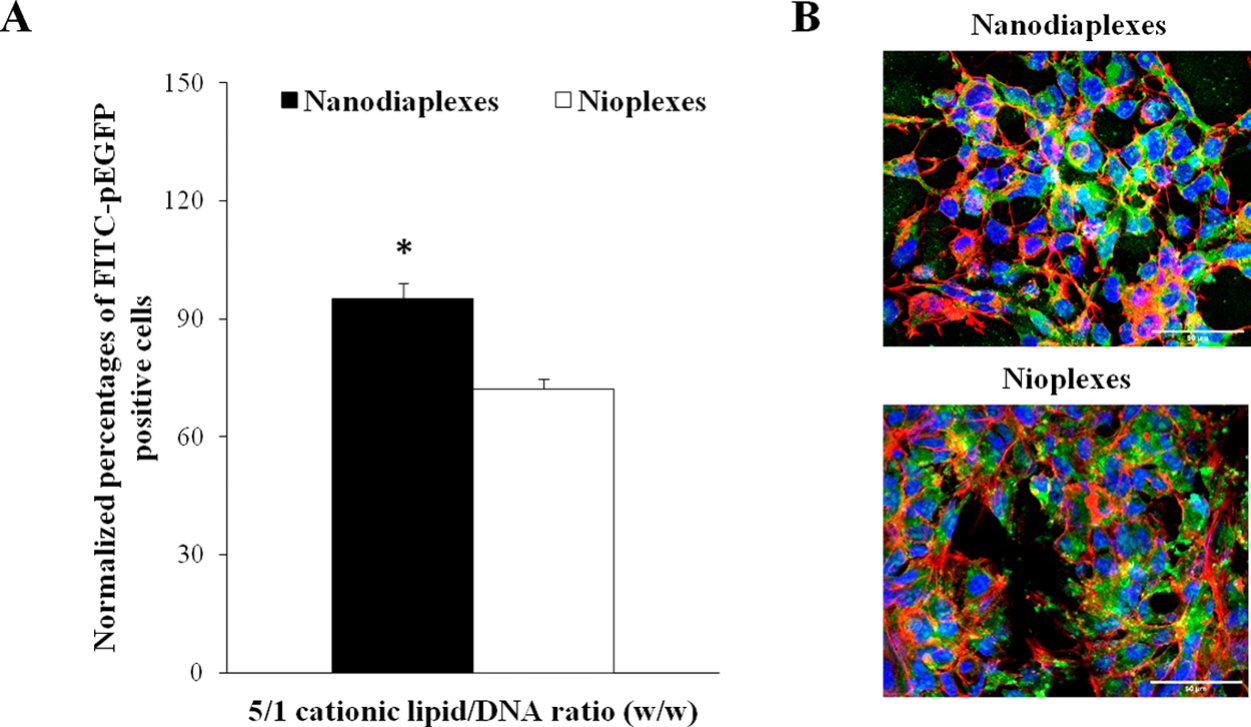
Cellular uptake of complexes at the 5/1 lipid/DNA ratio, analyzed 4 h after transfection in the HEK-293 cell line. (A) Normalized percentage of FITC-pEGFP positive cells. Each value represents the mean ± SD of three measurements; * *p* > 0.05 for nanodiaplexes vs nioplexes. (B) Confocal microscopy images showing the cellular uptake of nanodiaplexes and nioplexes. Cell nuclei were colored in blue (DAPI), F-actin in red (Phalloidin), and nanodiaplexes and nioplexes in green (FITC). Scale bar: 50 μm.

Cell internalization of nanodiaplexes at the 5/1 lipid/DNA ratio in the HEK-293 cell line 4 h after their exposure to these complexes showed significantly higher values of FITC-pEGFP positive signal than their counterpart nioplexes (95.1 ± 3.9 vs 72.2 ± 2.4%; *p* < 0.05) (Figure 6A). The positive control of transfection Lipofectamine 2000 showed 60% of FITC-pEGFP positive cells 4 h after the exposure of cells to lipoplexes (data not shown), and all data were normalized in relation to this percentage value. Representative confocal microscopy images exhibiting cellular uptake of nanodiaplexes and nioplexes at the 5/1 ratio are shown in Figure 6B.

### Intracellular Trafficking and Endosomal Escape

**Figure 7:**
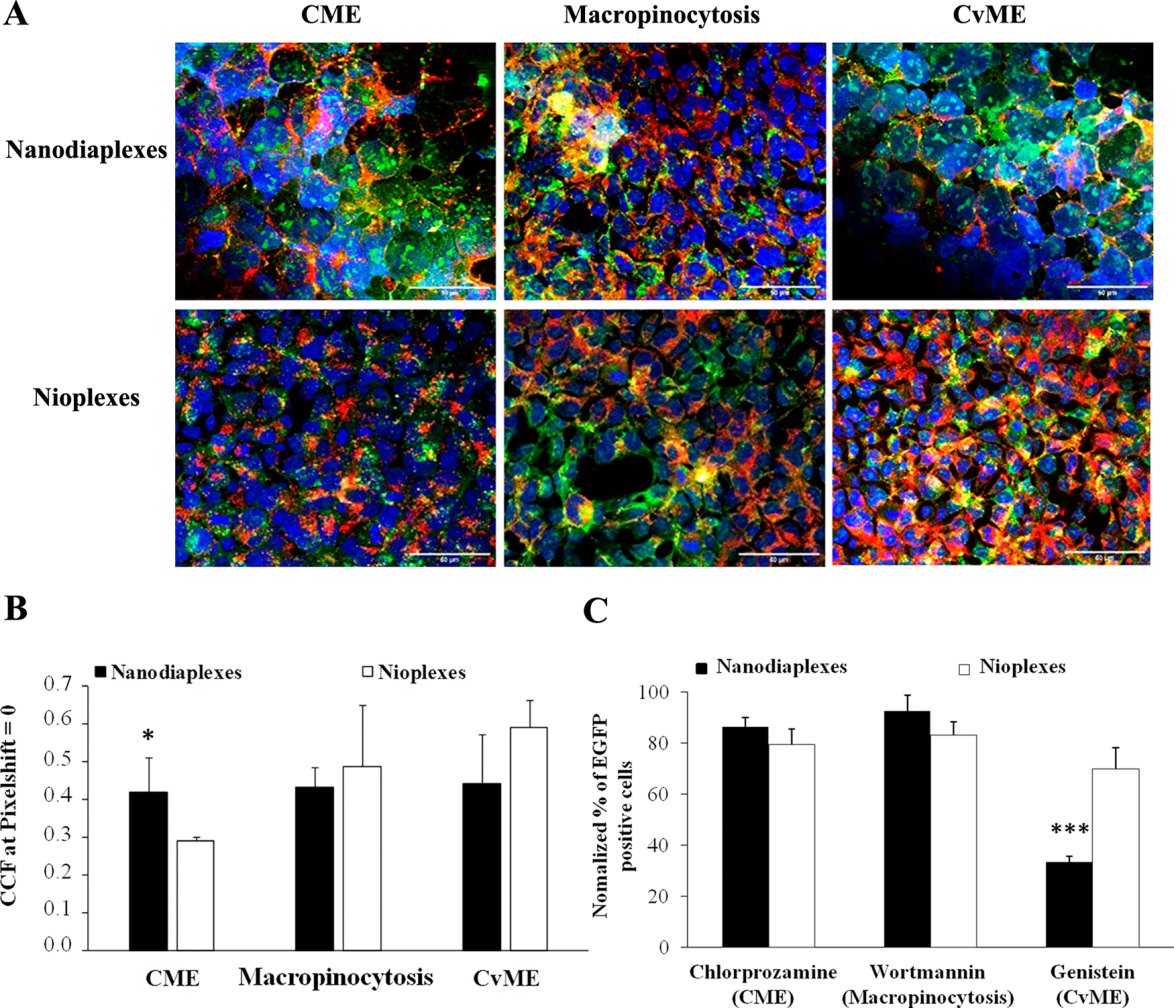
Intracellular disposition assay of nanodiaplexes and nioplexes in HEK-293 cells. (A) Qualitative analysis of colocalization by confocal microscopy. Scale bar: 50 μm. (B) Quantitative determination of colocalization by cross-correlation analysis. Data are represented as mean ± SD of three measurements; * *p* > 0.05 for nanodiaplexes vs nioplexes. (C) Transfection performance after the addition of specific endocytic inhibitors. The values were normalized to the transfection without an inhibitor. ***p < 0.001.

Representative images showing the colocalization of the complexes (green signal) with the intracellular pathway as early endosomes (red signal), either CME, macropinocytosis or CvME, can be observed in Figure 7A. Colocalization of red and green fluorescence signals led to yellow/orange dots.

The quantification of the colocalization signal for each formulation indicated that there was not an endocytic pathway that rose above the others (Figure 7B). However, it pointed out that the highest difference between nanodiaplexes and nioplexes was observed in the CME pathway (*p* < 0.05), where nanodiaplexes colocalized more than nioplexes in this pathway. Interestingly, regarding the involvement of each pathway in transfection efficiency, the selective inhibition of CvME (genistein) significantly decreased transfection efficiency mediated by nanodiaplexes (*p* < 0.001), while in the case of nioplexes, transfection efficiency was slightly affected, overall when clathrin and micropinocytosis were inhibited with chlorpromazine hydrochloride and wortmannin inhibitors, respectively (Figure 7C).

**Figure 8:**
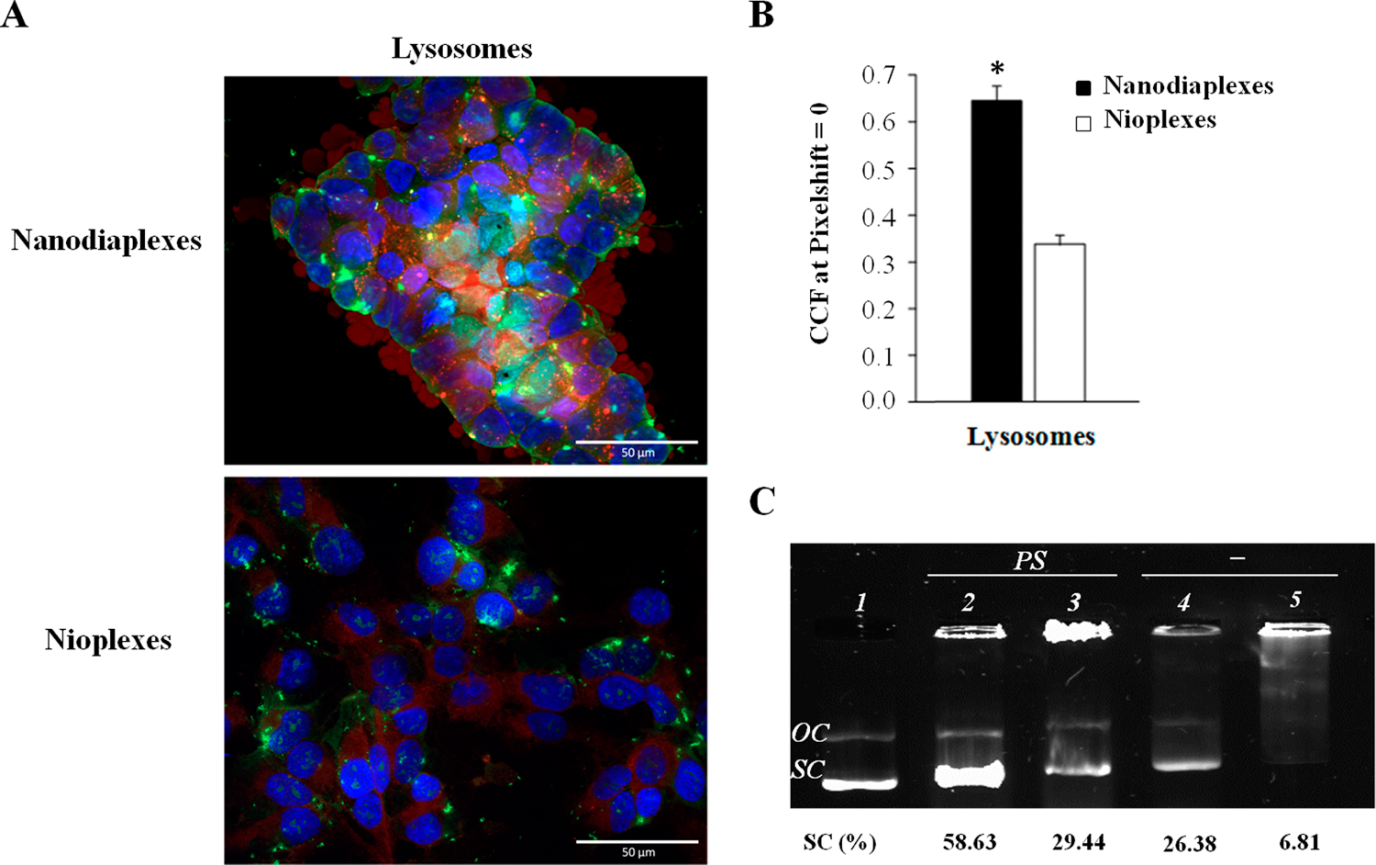
Biological performance of nanodiaplexes and nioplexes in lysosomes of HEK-293 cells. (A) Qualitative analysis of colocalization by confocal microscopy. (B) Quantitative determination of colocalization by cross-correlation analysis. Data are represented as mean ± SD of three measurements; * *p* > 0.05 for nanodiaplexes vs nioplexes. (C) DNA release profiles evaluated by gel electrophoresis. Lane 1, naked DNA; lane 2, nanodiaplexes incubated with PS; lane 3, nioplexes incubated with PS; lane 4, nanodiaplexes; lane 5, nioplexes. PS refers to phosphatidylserine micelles; OC: open circular form; SC: supercoiled form.

Following the trafficking of complexes along the cell to the late endosomes, further assays regarding the colocalization of the complexes (green signal) with lysosomes as the late endosomal compartment (red signal) were performed (Figure 8A). Data showed that nanodiaplexes colocalized more in lysosomes compared to nioplexes (*p* < 0.05) (Figure 8B). As observed in Figure 8C, in the case of nioplexes and in the absence of PS vesicles (lane 5), practically all DNA was retained, because the percentage of SC bands (the most bioactive form)^38,39^ only represented 6.81% of all DNA signals. However, in the case of nanodiaplexes (lane 4), the percentage of the SC signal increased to 26.38% of all DNA signals, which means that nioplexes showed a greater ability to bind DNA than nanodiaplexes. These results are in agreement with those obtained in Figure 4. However, when both complexes were coincubated with PS vesicles to evaluate endosomal escape properties, we observed a stronger SC signal in the case of nanodiaplexes (lane 2, 58.63% of all DNA signal) compared to nioplexes (lane 3, 29.44%). Consequently, the presence of NDs in the niosome formulation increased 1.4-fold times the endosomal escape properties, as can be deduced by subtracting the % of SC DNA signals of control lane 4 (26.38%) and lane 5 (6.81%) that correspond to nanodiaplexes and nioplexes, respectively, to the percentage of SC DNA signal on lane 2 (58.63%) and lane 3 (29.44%).

### Gene Delivery Capacity of Nanodiaplexes to Primary CNS Cells

**Figure 9:**
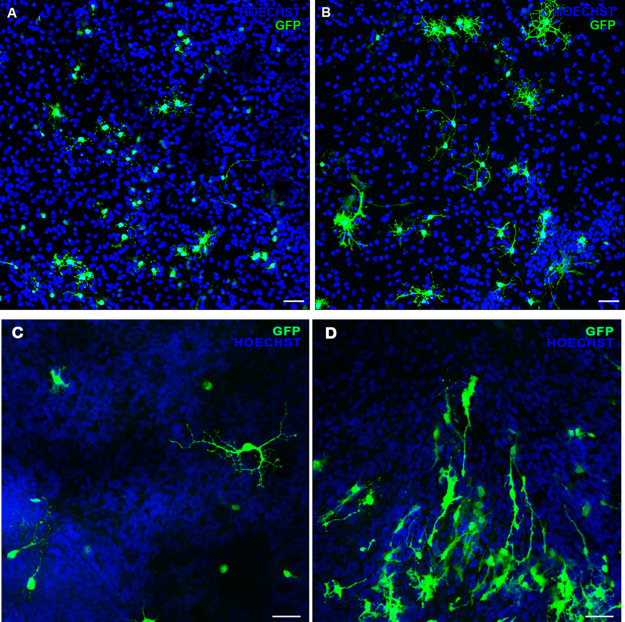
GFP expression in embrionary rat CNS primary cells. Neuronal and retinal primary cells transfected with nanodiaplexes (A,C) at the 5/1lipid/DNA ratio and the positive control Lipofectamine 2000 in primary neuronal (B) and retinal cells (D). Cell nuclei were stained with Hoechst 33342 (blue). Scale bar: 50 μm.

The assessment of the transfection process in primary CNS cells from cerebral (Figure 9A) and retinal (Figure 9C) cultures exposed to nanodiaplexes at the 5/1 ratio showed GFP signals in both cases, compared with Lipofectamine 2000 positive control in cerebral and retinal primary cells, respectively (Figure 9B,D). These results corroborate the gene delivery capacity of this vector into CNS cells.

## Discussion

The existence of carbon-based nanomaterials with promising features for gene therapy purposes emerges as an attractive strategy to improve nonviral transfection efficiency, moving ever closer to overcome the present translational barrier to biomedical applications. In this field of carbon nanostructures, such as nanotubes, graphene, and graphene oxide, NDs have gained momentum because of their particular geometrical characteristics, particular surface chemistry with high Young’s modulus and large scale production capability, as well as nontoxic and biocompatible properties.^40−42^ NDs are spherical shape structures with an average diameter of ∼5 nm accompanied by a low dispersity index and a relatively large surface area. One of the main issues of NDs is the tendency to self-agglomeration caused by their nanometer size and van der Waals forces, which also confers them a poor stability in a variety of media. In fact, a mean size of 89 nm was found for NDs alone in water suspension (Figure S1). Therefore, NDs must be functionalized or bound to other components, normally polymers^43^ for gene delivery purposes, although some drug delivery works have also bound NDs to liposome phospholipids.^44,45^ In this regard, NDs have been used in gene therapy by their single binding to specific polymers which confers them the ability to bind and deliver the genetic material.^11−15^

Another state-of-the-art nonviral approach is the use of niosomes which are cationic lipid-based vesicles with nonionic surfactants widely used in gene therapy and are gaining interest over liposomes because of their lower cost, higher biocompatibility, and stability.^20,46^ Taking into account the natural ability of niosomes for high genetic material containment and high biocompatibility of both NDs and niosomes, here we propose a promising novel gene therapy strategy combining their attractive features to burst a powerful nanoplatform with high transfection efficiency and biocompatibility. In particular, we elaborated the NDs integrated into cationic niosomes, named nanodiasomes, composed of DOTMA as cationic lipids and polysorbate 20 as a nonionic surfactant.

In a first step, we optimized, in terms of physicochemical and biological properties, a nanodiasome formulation employing different ND/DOTMA mass ratios (Supporting Information, Figures S1 and S2). Interestingly, the more amount of DOTMA in the formulation, the smaller was the nanodiasome size and, consequently, it caused a slight increase of zeta potential.^47^ This decrease of the nanoparticle size could be explained by the electrostatic interactions between the positive charge of the cationic lipid and the negative charge of NDs where the physical and chemical properties of NDs would promote an increasing degree of compaction of the nanoparticle. Thus, the more cationic lipid, the higher electrostatic interactions with NDs, reducing the final size of the nanoparticle. In this sense, some studies have observed that different doses of NDs might adsorb to the surface of the lipid membrane of liposomes without affecting the packing of the bilayer.^45^ Hence, it could be suggested that the cationic lipid amount in the formulation is responsible for the physicochemical changes in the nanodiasome. Even though the increase of cationic lipids can promote cell death because of the positive charge that it confers to the vector, the amount of DOTMA—from 1.1 to 3.7 mM—used in these formulations presented good biocompatibility in all cases. In general terms, cell viability values were around 95% at 2/1 and 5/1 lipid/DNA ratios, while slightly progressive cytotoxicity was observed at 10/1 and 15/1 ratios diminishing cell viability to 85% (Figure S1). We found an optimum balance between biocompatibility and transfection efficiency employing NDT12 at 5/1 lipid/DNA ratios, so further studies were carried out with this nanodiasome formulation.

To explore the influence of NDs integrated as a helper component into niosomes, additional physicochemical, transfection, biocompatibility, and intracellular trafficking analyses were carried out with NDT12 nanodiasomes compared to the same formulation devoid of NDs. Concerning the physicochemical parameters (Figure 2), nanodiasomes and nanodiaplexes presented nearly a 30% higher particle size than niosomes and nioplexes because of the ND content, maintaining in all cases mean diameter sizes below 200 nm. As expected, sizes increased when complexing the formulations with the plasmid genetic material, while zeta potential decreased because of the neutralization of positive and negative charges.^48^ Some mean dispersity values were slightly high, as is the case of niosomes/nioplexes and nanodiaplexes at the 5/1 ratio (Figure 2B), which could be due to the presence of few aggregates in the sample, denoted by the presence of a high peak in the particle size-distribution intensity at the micrometer scale (Figure 2C,D). Overall, mean dispersity values were lower in nanodiasomes and nanodiaplexes, pointing out to a better homogeneity of this formulation compared to niosomes. In fact, TEM captures revealed a clear spherical morphology of nanodiasomes with no aggregations (Figure 3A), where ND particles are integrated into the lipid layer of this nonviral vector (Figure 3B,C). Therefore, although both formulations showed suitable characteristics for gene therapy purposes, they presented some physicochemical variations among them.

After physicochemical characterization, in vitro transfection studies were carried out in the HEK-293 cell line, which is considered a good model for transfection. Transfection efficiency of nanodiaplexes was much greater than that of nioplexes at all lipid/DNA ratios, overall at the 5/1 ratio where 75% increment was observed when compared to its counterpart niosomes devoid of NDs (Figure 5). Of note, this higher transfection efficiency was also accompanied by higher cell viability values around 90%. In this regard, the 1.25 mg/mL concentration of NDs employed in the present study is higher than that described in other studies—ranging from 0.01 to 1 mg/mL—for maintaining a suitable biocompatibility.^49^ Hence, these observations highlight the benefits of combining a highly biocompatible material presenting high adsorption properties, such as NDs, with other highly biocompatible, stable, and high-loading capacity vectors such as cationic niosomes.

Cellular uptake is one of the most decisive criteria to be considered when evaluating the delivery of the cargo into cells. The 25% increase in the cellular uptake of nanodiaplexes vs nioplexes could be considered as one of the potential factors that enhanced their transfection efficiency (Figure 6), in accordance with previous reports where NDs increased the cellular uptake of zwitterionic liposomes for drug delivery purposes.^45^ In this regard, relevant physicochemical parameters of nonviral vectors, such as size, zeta potential, shape, and rigidity seem to affect the internalization process and posterior intracellular pathway followed by the nanoparticle and the genetic material. In fact, rigid structures along with small size and positive zeta potential values may be the most favorable features to enhance cellular uptake.^50^ In this sense, and taking into account that nanodiaplexes are bigger and slightly more positive than nioplexes, the physical and chemical properties of NDs could contribute to the rigidity of the vector and therefore promote the cell internalization of nanodiaplexes.

Additionally, the internalization pathway followed by the vector and its DNA can be critical to its intracellular fate. Most of the nanoparticles, including the lipid-based vectors, are internalized by pinocytosis, principally through receptor-mediated endocytosis.^50^ In this work, we did not observe a predominant endocytic pathway when using nanodiaplexes or nioplexes, but our data suggested that the first ones trafficked more by CME than nioplexes (Figure 7,BA). In the case of specific endocytic pathway inhibition studies, our data revealed that when nanodiaplexes were administered to HEK-293 cells, endocytosis mediated by caveolae was the most efficient endocytic pathway to transfect cells, because when this pathway was inhibited by genistein, the percentage of cells expressing EGFP plasmid decreased to around 30% value (Figure 7C). Consequently, the transfection performance of NDs integrated into niosome formulations as nonviral vectors could be promoted by the addition of chemical components that induce the CvME pathway.^51^ However, the inhibition of CME and macropinocytosis only decreased transfection efficiency values to around 90% values. In the case of transfection efficiency mediated by nioplexes, the percentage of transfected cells decreased only to around 80–70% values with the use of the three cellular uptake inhibitors, which suggest that probably other endocytic pathways could be playing a more relevant role in the complex transfection process.^52,53^

Further trafficking studies extending to the late endosomal compartment were carried out. It was observed that nanodiaplexes colocalized more with lysosomes than nioplexes (Figure 8,BA), pointing out that NDs might promote the CME pathway, and the internalized vesicle would lose the clathrin coat obtaining an early endosome that turns into a late endosome which becomes a lysosome.^54^ These results are in accordance with a previous but more basic intracellular trafficking study of fluorescent NDs which reported their internalization in early endosomes followed by lysosomal localization. Interestingly, authors explained the lysosomal compartment as a previous step for the exocytosis of these fluorescent NDs via the lysosomal degradation pathway.^55^ Of note, the artificial anionic micelles of PS developed in the present research work to mimic this late endosomal compartment (Figure 8C) revealed that nanodiasomes had better endosomal escape properties than niosomes. After subtracting the % of the control SC DNA signal observed in lanes 4 (26.38%, nanodiaplexes) and 5 (6.81%, nioplexes) from the % of SC DNA signal observed in lanes 2 and 3 (58.63%, nanodiaplexes and 29.44%, nioplexes, respectively, coincubated with PS vesicles), the obtained value was 32.26% for nanodiaplexes and 22.63% for nioplexes. Such results suggest that the presence of NDs in the formulation increases 1.43-fold (around 30%) the endosomal escape property in HEK-293 cells than the formulation without NDs. Among the mechanisms by which nanodiaplexes could escape the endosomes, the most likely one consists of the direct fusion of the nanoparticles with the endosome membrane, as shown by their colocalization with lysosomes, along with the creation of pores in the endosome surface caused by the induction of stress and internal tension in the membrane, as evidenced by the great DNA released from this kind of compartment.^56^ Taken all together, our data suggest that the enhanced transfection efficiency of nanodiaplexes over nioplexes might be attributed mainly to the higher cellular uptake, probably due to the rigidity that NDs confer to nanodiaplexes, and to the lysosomal escape properties promoted by NDs.

Additional gene delivery studies in primary CNS cells from cerebral and retinal sources were carried out with nanodiaplexes at the 5/1 lipid/DNA ratio to move on a more realistic and translational microenvironment of an in vivo model. Immunocytochemistry showed the GFP signal in both primary cell cultures (Figure 9A,C), pointing out to the capacity of nanodiaplexes to successfully deliver genetic materials to CNS cells. Because CNS diseases constitute an area where the development of new therapeutic strategies represents a burning need,^57^ the emerging role of NDs in niosomes for gene delivery applications represents a major finding. In addition, presumably NDs and not other carbon-based nanomaterials would possess excellent compatibility with biological systems, resulting in an encouraging candidate for biomedical applications.^49^ In this sense, the nontoxicity of NDs after their in vivo administration by intratracheal instillation, which is a decisive route when analyzing the potential toxic effect of nanocarriers on respiratory system, has been described.^42^ Although promising, these results represent a proof-of-concept and further studies in animal models would be required for corroborating the observed potential of nanodiasomes for gene delivery.

## Conclusions

The main findings are the following ones: (1) NDs can be integrated into niosome formulations as helper components, maintaining suitable physicochemical characteristics for gene delivery; (2) niosomes with NDs represent a novel nonviral vector that binds, releases, and protects the genetic material from degradation; (3) niosomes containing NDs present higher biocompatibility and transfection efficiency in vitro than those devoid of NDs, mainly explained by the higher cellular uptake promoted by NDs; (4) NDs integrated into niosomes are involved in lysosomal escape; and (5) these nanodiasomes can deliver genetic materials to primary CNS cells. Hence, NDs integrated into niosomes emerge as a powerful nanoplatform for gene therapy purposes, especially for CNS disorders, and may constitute a promising and safe nonviral strategy with potential biomedical applications.

## Supporting Information Available

Additional experimental details and results, including multimedia content.
Biophysical screening of the nanodiasome formulations. Tomogram reconstruction and volumetric representation (PDF)
Figure S1: Physicochemical characterization of nanodiasome formulations (PDF)
Figure S2: Normalized percentages of EGFP-positive live cells and cell viability in HEK-293 cell line 48 hours post-transfection with nanodiaplexes. (PDF)
Cryo-tomography reconstruction and the volumetric representation of the tomograms (AVI) (MP4)

## Supplementary Material

am2c02182_si_003.pdf

am2c02182_si_004.avi

## Abbreviations

CCF: cross-correlation function
CME: clathrin-mediated endocytosis
CNS: central nervous system
CvME: caveolae mediated endocytosis
*Đ*: dispersity index
DCM: dichloromethane
EMEM: minimal essential medium
DOTMA: 1,2-di-O-octadecenyl-3-trimethylammonium propane chloride salt
EGFP: enhanced green fluorescent protein
FBS: fetal bovine serum
FITC: fluorescein isothiocyanate
HEK-293: human embryonic kidney cells
MFI: mean fluorescence intensity
ND: nanodiamond
NDT: ND/DOTMA mass ratio
PBS: phosphate buffered saline
PS: phosphatidylserine
RT: room temperature
SDS: sodium dodecyl sulfate
TEM: transmission electron microscopy
