# Correction to “Nanodiamonds Integration into Niosomes as an Emerging and Efficient Gene Therapy Nanoplatform for Central Nervous System Diseases”

**DOI:** 10.1021/acsami.2c18984

**Published:** 2023-01-10

**Authors:** Nuseibah AL Qtaish, Idoia Gallego, Alejandro J. Paredes, Ilia Villate-Beitia, Cristina Soto-Sánchez, Gema Martínez-Navarrete, Myriam Sainz-Ramos, Tania B. Lopez-Mendez, Francisco Javier Chichón, Noelia Zamarreño, Eduardo Fernández, Gustavo Puras, José Luis Pedraz

**Affiliations:** † NanoBioCel Research Group, Laboratory of Pharmacy and Pharmaceutical Technology, Faculty of Pharmacy, University of the Basque Country (UPV/EHU), Paseo de la Universidad 7, 01006 Vitoria-Gasteiz, Spain; ‡ Networking Research Centre of Bioengineering, Biomaterials and Nanomedicine (CIBER-BBN), Institute of Health Carlos III, 28029 Madrid, Spain; § Bioaraba, NanoBioCel Research Group, 01009 Vitoria-Gasteiz, Spain; ∥ Research and Development Unit in Pharmaceutical Technology (UNITEFA), CONICET and Department of Pharmaceutical Sciences, Chemistry Sciences Faculty, National University of Córdoba, Haya de la Torre y Medina Allende, X5000XHUA Córdoba, Argentina; ⊥ School of Pharmacy, Queen’s University Belfast, Medical Biology Centre, 97 Lisburn Road, Belfast BT9 7BL, Northern Ireland, U.K.; # Neuroprothesis and Neuroengineering Research Group, Institute of Bioengineering, Miguel Hernández University, Avenida de la Universidad, 03202 Elche, Spain; ¶ CryoEM CSIC Facility, Structure of Macromolecules Department, Centro Nacional de Biotecnología (CNB-CSIC), Calle Darwin no. 3, 28049 Madrid, Spain

In the original version of this article, the authors Francisco Javier Chichón and Noelia Zamarreño were incorrectly not included in the authorship. This Correction contains the corrected Authorship, Author Information (affiliations), Author Contributions, Acknowledgments, and Supporting Information. The changes do not alter the major conclusions of this work.

## Supporting Information Available

Biophysical screening of the nanodiasome formulations composed of niosomes with nanodiamonds as helper component, physicochemical characterization of nanodiasomes at different ND/DOTMA mass ratios (including Figure S1 showing the data), transfection efficiency of nanodiasomes at different ND/DOTMA mass ratios, Figure S2 showing normalized percentages of EGFP positive live cells and cell viability, and description of the videos (PDF)

