# Supplementary figures and images for: Nanodiamond Integration into Niosomes as an Emerging and Efficient Gene Therapy Nanoplatform for Central Nervous System Diseases
Source: ACS Appl Mater Interfaces. 2022 Mar 15;14(11):13665–77. doi: 10.1021/acsami.2c02182 (PMC8949757; doi:10.1021/acsami.2c02182)

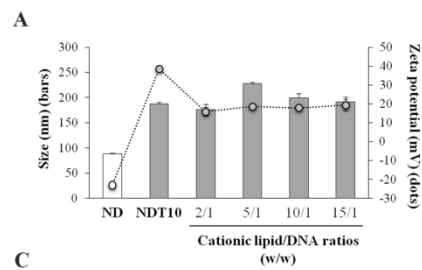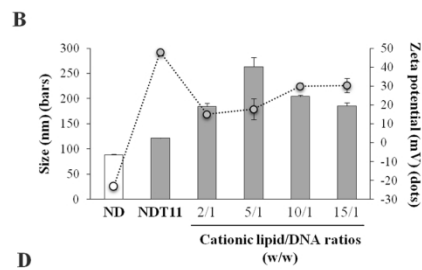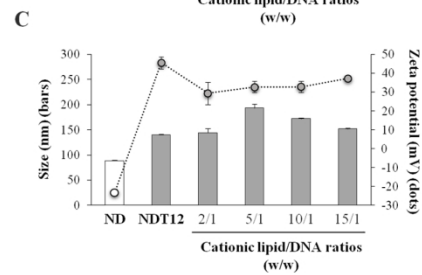

**D**

|                                 |       | ND   |      | NDT10 |      | NDT11 |      | NDT12 |      |
|---------------------------------|-------|------|------|-------|------|-------|------|-------|------|
|                                 |       | Mean | SD   | Mean  | SD   | Mean  | SD   | Mean  | SD   |
| Cationic lipid/DNA ratios (w/w) | Alone | 0.28 | 0.02 | 0.18  | 0.02 | 0.18  | 0.01 | 0.21  | 0.08 |
|                                 | 2/1   |      |      | 0.32  | 0.01 | 0.35  | 0.03 | 0.33  | 0.02 |
|                                 | 5/1   |      |      | 0.30  | 0.04 | 0.35  | 0.04 | 0.37  | 0.03 |
|                                 | 10/1  |      |      | 0.30  | 0.01 | 0.35  | 0.01 | 0.35  | 0.04 |
|                                 | 15/1  |      |      | 0.29  | 0.03 | 0.33  | 0.01 | 0.35  | 0.01 |

Supplement: Supplementary file 2 — am2c02182_si_002.pdf [file am2c02182_si_002.pdf]

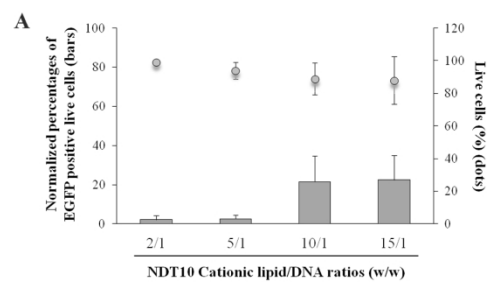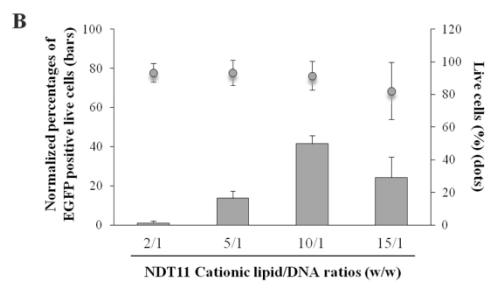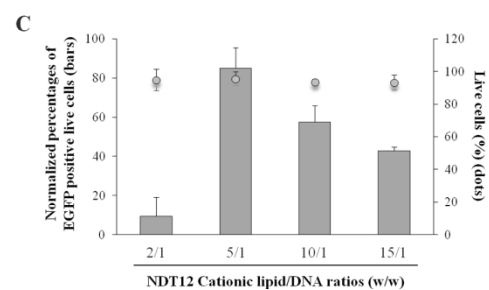

Supplement: Supplementary file 3 — am2c02182_si_003.pdf [file am2c02182_si_003.pdf]
