# Supplementary material for: Correction to “Nanodiamonds Integration into Niosomes as an Emerging and Efficient Gene Therapy Nanoplatform for Central Nervous System Diseases”
Source: ACS Appl Mater Interfaces. 2023 Jan 10;15(3):4857–8. doi: 10.1021/acsami.2c18984 (PMC9880947; doi:10.1021/acsami.2c18984)
Supplement: Supplementary file 1 — am2c18984_si_001.pdf [file am2c18984_si_001.pdf]

**Supporting Information**

# Nanodiamonds Integration into Niosomes as an Emerging and Efficient Gene Therapy Nanoplatform for Central Nervous System Diseases

*Nuseibah AL Qtaish[a,b,‡], Idoia Gallego[a,b,c,‡], Alejandro J. Paredes[d,e], Ilia Villate- Beitia[a,b,c], Cristina Soto-Sánchez[b,f], Gema Martínez-Navarrete[b,f], Myriam Sainz-Ramos[a,b,c], Tania B. Lopez-Mendez[a,b,c], Francisco Javier Chichón[g], Noelia Zamarreño[g], Eduardo Fernández[b,f], Gustavo Puras[a,b,c,*], and José Luis Pedraz[a,b,c,*].*

[a] NanoBioCel Research Group, Laboratory of Pharmacy and Pharmaceutical Technology. Faculty of Pharmacy, University of the Basque Country (UPV/EHU), Paseo de la Universidad 7, 01006 Vitoria-Gasteiz, Spain.

[b] Networking Research Centre of Bioengineering, Biomaterials and Nanomedicine (CIBER-BBN), Institute of Health Carlos III, 28029 Madrid, Spain

[c] Bioaraba, NanoBioCel Research Group, 01009 Vitoria-Gasteiz, Spain.

[d] Research and Development Unit in Pharmaceutical Technology (UNITEFA), CONICET and Department of Pharmaceutical Sciences, Chemistry Sciences Faculty, National University of Córdoba. Haya de la Torre y Medina Allende, X5000XHUA, Córdoba, Argentina.

[e] School of Pharmacy, Queen's University Belfast, Medical Biology Centre, 97 Lisburn Road, Belfast, BT9 7BL, Northern Ireland, UK.

[f] Neuroprothesis and Neuroengineering Research Group, Institute of Bioengineering, Miguel Hernández University, Avenida de la Universidad, 03202 Elche, Spain.

[g] CryoEM CSIC Facility. Centro Nacional de Biotecnología. Calle Darwin n°3, 28049 Madrid, Spain.

‡These authors contributed equally

*Correspondence: address all correspondence to: joseluis.pedraz@ehu.eus and gustavo.puras@ehu.eus
(José Luis Pedraz +34945013091, Gustavo Puras +34945014536)

**Address of corresponding author**:
Professor José Luis Pedraz, PhD; Laboratory of Pharmacy and Pharmaceutical Technology.

Faculty of Pharmacy, University of the Basque Country (UPV/EHU)

Paseo de la Universidad 7, 01006 Vitoria-Gasteiz, Spain.

Phone number: +34945013091. Fax number: +34945013040.

E-mail address: joseluis.pedraz@ehu.eus

## 1. Biophysical screening of the nanodiasome formulations composed of niosomes with nanodiamonds as helper component

### EXPERIMENTAL SECTION

All the formulations were elaborated by the oil in water technique. The components employed for the study of the best balance NDs/DOTMA for the development of a suitable nanodiasome formulation for gene therapy purposes were: 250 µl of NDs (10 mg/ml in $H_2O$) ultrasonicated for 30 minutes and mixed with 2 ml of 0.5% Tween 20 and 1.75 ml of MilliQ water, as the aqueous phase. For the organic phase 1.25, 2.5 or 5 mg of the cationic lipid DOTMA were accurately weighted to obtain 1/0.5, 1/1 and 1/2 ND/DOTMA mass ratios, respectively. The DOTMA was diluted in 1 ml of dichloromethane (DCM). The organic phase was added upon the aqueous phase and immediately sonicated for 30 minutes at 50 W. DCM was evaporated for 2 h at room temperature under magnetic stirring obtaining formulations named as nanodiasomes NDT10, NDT11 and NDT12, for ND/DOTMA at 1/0.5, 1/1 and 1/2 mass ratios, respectively.

## RESULTS

### Physicochemical characterization of nanodiasomes at different ND/DOTMA mass ratios

The average particle size of ND alone was 89 nm (Figure S1 A-C, white bar). This value increased when ND were integrated as helper component into niosomes to obtain nanodiasomes, which presented size values below 200 nm in all cases (Figure S1A-C, grey bars). The quantity of DOTMA in nanodiasomes had a faint influence on particle size, decreasing around 30% along with the increasing amounts of DOTMA in the formulations (NDT10: 187 nm; NDT11: 121 nm; NDT12: 140 nm) (Figure S1 A-C, grey bars). In general terms, when complexing with DNA, average particle size peaked at 5/1 ratio reaching 228 nm for NDT10, 264 nm for NDT11 and 194 nm for NDT12, which corresponded to 1.2-fold, 2.2-fold and 1.4-fold increase compared with their respective nanodiasomes. Then, the nanodiaplexes size decreased gradually at 10/1 and 15/1 cationic lipid/DNA mass ratio.

Zeta potential of ND alone was -23 mV (Figure S1 A-C, white dot) while it turned into positive to values between +38 mV and + 48 mV when ND were integrated into the niosomes as helper component (Figure S1 A-C, grey dots). In particular, zeta potential values increased gradually with the increasing amounts of cationic lipid in the formulation. Upon the addition of pEGFP to nanodiasomes, zeta potential values decreased at 2/1 cationic lipid/DNA mass ratio and increased gradually with the increasing ratios. In all cases, zeta potential of nanodiaplexes remained positive between +15 mV and +37 mV.

Mean dispersity values obtained for nanodiasomes remained stable in all cases, between 0.18 and 0.21, and were lower than the ones corresponding to ND alone (0.28) (Figure S1D). Complexation of nanodiasomes with DNA conducted to slight changes in this parameter, presenting values below 0.4 in all cases.

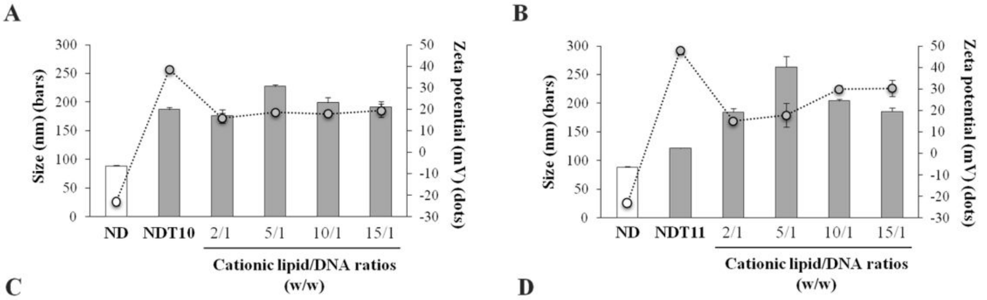


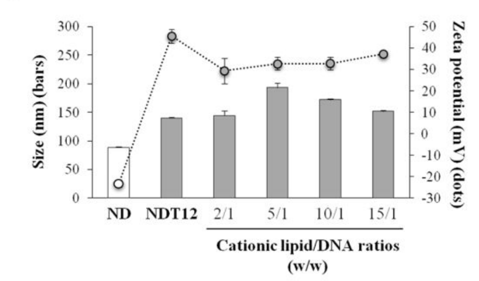


D

| | | ND | | NDT10 | | NDT11 | | NDT12 | |
|---|---|---|---|---|---|---|---|---|---|
| | | Mean Đ | SD | Mean Đ | SD | Mean Đ | SD | Mean Đ | SD |
| Alone | | 0.28 | 0.02 | 0.18 | 0.02 | 0.18 | 0.01 | 0.21 | 0.08 |
| Cationic lipid/DNA ratios (w/w) | 2/1 | | | 0.32 | 0.01 | 0.35 | 0.03 | 0.33 | 0.02 |
| | 5/1 | | | 0.30 | 0.04 | 0.35 | 0.04 | 0.37 | 0.03 |
| | 10/1 | | | 0.30 | 0.01 | 0.35 | 0.01 | 0.35 | 0.04 |
| | 15/1 | | | 0.29 | 0.03 | 0.33 | 0.01 | 0.35 | 0.01 |

**Figure S1:** Physicochemical characterization of nanodiasome formulations, elaborated with ND as helper component at different ND/DOTMA mass ratios (1/0.5, named NDT10; 1/1, named NDT11 and 1/2, named NDT12), and nanodiaplexes at different DOTMA/DNA mass ratios (2/1, 5/1, 10/1 and 15/1). **A-C.** Size (bars) and zeta potential (dots) for (A) NDT10 and corresponding nanodiaplexes (B) NDT11 and corresponding nanodiaplexes, and (C) NDT12 and corresponding nanodiaplexes. **D.** Dispersity and SD values of nanodiamonds, nanodiasomes and nanodiaplexes. Each value represents the mean ± standard deviation of three measurements. ND, means nanodiamonds; Đ, means dispersity.

## Transfection efficiency of nanodiasomes at different ND/DOTMA mass ratios

Analysis of EGFP expression in living cells, after transfecting with the complexes based on the three nanodiasome formulations, clearly showed a high transfection efficiency employing NDT12 formulation at 5/1 cationic lipid/DNA mass ratio (Figure S2, bars). In particular, for NDT10 the maximum value obtained was around 20% of EGFP expression at 10/1 and 15/1 ratios (Figure S2 A, bars); for NDT11 this parameter doubled to 40% at 10/1 ratio (Figure S2 B, bars) and in the case of NDT12 it doubled over again the transfection efficiency presenting more than 85% of living cells expressing EGFP at 5/1 ratio (Figure S2 C, bars). Additionally, the biocompatibility of NDT12 was higher and remained constant at all cationic lipid/DNA mass ratios, compared with NDT10 and NDT11 (Figure S2, dots). In general terms, cell viability for NDT10 and NDT11 was around 95% at 2/1 ratio but declined progressively by 12% at 15/1 ratio (Figure S2 A and B, respectively, dots), while in the case of NDT12 cell viability was around 95% at all ratios (Figure S2 C, dots). Lipofectamine at 2/1 ratio was employed as a positive control for transfection, which presented a 43% of EGFP expression in live cells and 81% of cell viability (data not shown). Transfection data were normalized in regard to this transfection value.

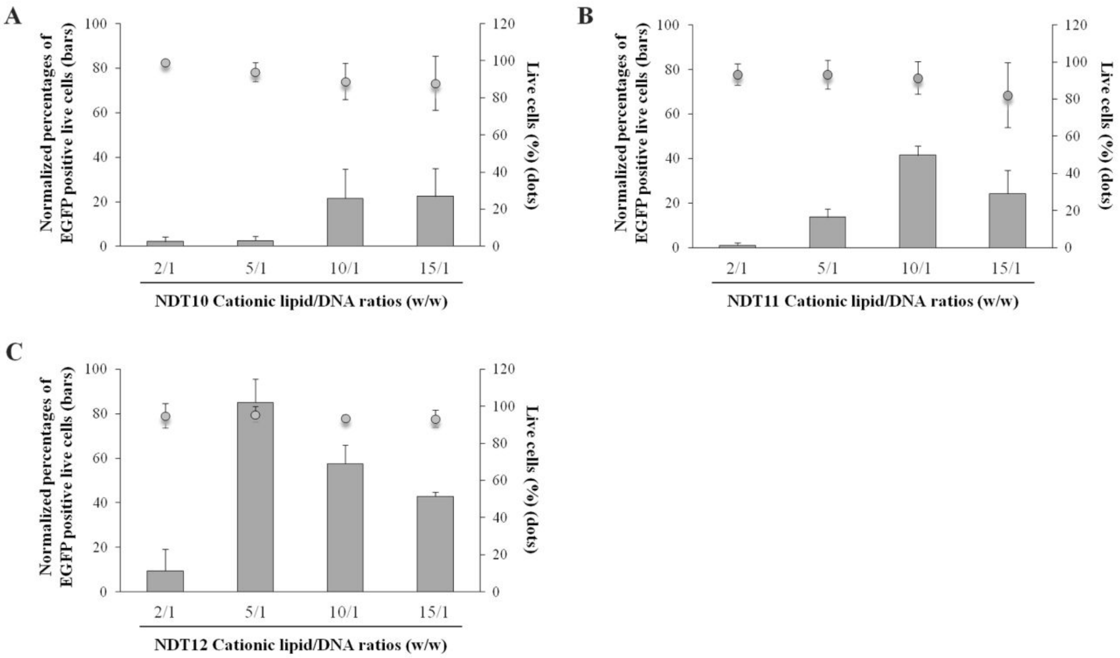


**Figure S2**: Normalized percentages of EGFP positive live cells (bars) and cell viability (dots) in HEK-293 cell line 48 hours post-transfection with nanodiaplexes. **A.** NDT10 nanodiaplexes **B.** NDT11 nanodiaplexes. **C.** NDT12 nanodiaplexes. Each value represents the mean ± standard deviation of n ≥ 4.

Therefore, from this biophysical screening to analyze the ideal ND/DOTMA balance for the development of a good gene therapy vector, NDT12 met the best conditions as it presented suitable physiscochemical properties for gene delivery purposes and showed promising high values of transfection efficiency accompanied by great biocompatibility in HEK-293 cell line.

## 2. Additional multimedia supporting information

Tomo_11_3DS30_rec-1.mrc kept stack.avi (Lí

**Video S1:** Tomogram reconstruction showing the enclosed nano-diamonds. Scale bar represent 100 nm.

Tomo2_movie.mp4

**Video S2:** Volumetric representation of the tomograms. Higher densities of the tomogram (more electron-dense material) corresponds to gold nanoparticles added to the sample for tilt series alignment (yellow). Medium densities are labeled in cyan and are linked to nanodiamonds (turquoise blue).
